# Progranulin inhibits expression and release of chemokines CXCL9 and CXCL10 in a TNFR1 dependent manner

**DOI:** 10.1038/srep21115

**Published:** 2016-02-19

**Authors:** Jyoti Joshi Mundra, Jinlong Jian, Priyal Bhagat, Chuan-ju Liu

**Affiliations:** 1 Department of Orthopaedic Surgery, New York University School of Medicine, New York, NY10003, USA; 2 Touro College of Medicine New York, New York 10016, USA; 3 Department of Cell Biology, New York University School of Medicine, New York, New York 10016, USA

## Abstract

Progranulin (PGRN), a pleiotrophic growth factor, is known to play an important role in the maintenance and regulation of the homeostatic dynamics of normal tissue development, proliferation, regeneration, and host-defense. PGRN also has potent anti-inflammatory functionality, and deregulated PGRN is associated with rheumatoid arthritis and inflammatory bowel disease. We have previously reported that PGRN directly binds to TNFR and significantly enhances T_reg_ population and stimulatesIL-10 production. To further investigate PGRN’s function in the immune system we performed a gene array analysis on CD4+ T cells from wild type B6 mice and PGRN −/− mice. We identified many chemokines and their receptors, among which CXCL9 and CXCL10 were most prominent, that were significantly induced in PGRN null mice. Administration of recombinant PGRN protein strongly inhibited TNF and IFN-γ-induced CXCL9 and CXCL10 expression. In addition, CXCL9 expression is strongly upregulated in PGRN KO mice and its level is correlated with severity of inflammation in a dermatitis model. Further, we have demonstrated that PGRN-mediated inhibition of chemokine expression largely depends on TNFR1. Taken together, this study provides new insights into the mechanisms underlying PGRN mediated regulation of various inflammatory and autoimmune diseases.

Progranulin (PGRN) is a pluripotent, ubiquitously expressed secretory growth factor, especially abundant in cells of the epithelium, central nervous system and immune system. PGRN is an integral factor in multiple physiological and pathological processes, such as wound healing^1^, tumorigenesis^2^, and immune response^3^. Mutations of the progranulin (*GRN*) gene have been linked to frontal temple lobe dementia^4^,^5^, as well as many other neurodegenerative diseases^6^. PGRN has also shown potent anti-inflammatory effects in various inflammatory models^7^,^8^,^9^,^10^,^11^,^12^,^13^,^14^,^15^.

We previously established that PGRN binds to TNF receptors (TNFR), and have shown that PGRN-deficient mice are susceptible to collagen-induced arthritis (CIA) and inflammatory bowel disease (IBD)^12^,^13^. Moreover, we observed that PGRN significantly upregulates T_reg_ population and induces IL-10 production^12^,^13^,^16^. Additional reports have substantiated the association between PGRN and systemic inflammation and autoimmunity^17^,^18^,^19^,^20^. Therefore, to better understand how PGRN influences anti-inflammatory activity in the immune system, we performed a whole genome array on CD4^+^ T cells isolated from wild type B6 mice and PGRN null (−/−) B6 mice. We identified that several proinflammatory chemokines and their receptors, among which CXCL9 and CXCL10 were most significant, were upregulated in PGRN^−/−^ mice.

Chemokines are a group of small proteins which stimulate leukocyte recruitment to inflammatory sites and enable the clearance of foreign pathogens. Deregulated production of chemokines leads to the development of autoimmune disorders^21^. Chemokines have been classified into four families depending on the position of the N-terminal cysteine. The largest group of chemokines, CC chemokines, has the first 2 cysteine in an adjacent position and the second largest group, CXC chemokines, has the first 2 of 4 total cysteine separated by an intervening amino acid. CXCL9 and CXCL10 belong to the CXC chemokine family and have been found to be elevated in sera, synovial fluid, and synovial tissue of rheumatoid arthritis (RA) patients^22^,^23^. Higher serum levels of CXCL9 and CXCL10 are associated with more severe RA as compared to controls and reduction in the serum concentration of these chemokines is associated with RA rehabilitation. Accordingly, serum concentrations of CXCL9 and CXCL10 have been suggested as clinical biomarkers for RA disease activity^24^,^25^,^26^. Data from animal models of RA parallel these observations; serum and tissue levels of CXCL10 were also elevated in arthritic mice induced under the well-established collagen-induced arthritis (CIA) model^27^.

In this report we show that PGRN knockout (KO) mice have elevated levels of CXCL9 and CXCL10 and recombinant PGRN was able to inhibit expression and release of CXCL9 and CXCL10 *in vitro*. Moreover, CXCL9 is significantly induced in PGRN KO mice in a contact dermatitis model. Finally, we demonstrate that PGRN inhibition of CXCL9 and CXCL10 occurs mainly through TNFR1.

## Materials and Methods

### Gene expression analysis

All mice were housed and fed at NYU Animal Facility and the experimental design was approved by Institutional Animal Care and Use Committee. The experiments were carried out in accordance with the approved guidelines and regulations. CD4+ T cells were isolated from spleen of WT and PGRN−/− B6 mice using the Magcellect Naïve CD4+ T cell isolation kit (R&D Systems Minneapolis, MN). RNA was isolated following a 24 hour activation using CD3 and CD28 antibody (Ab). RNA quantity and quality were measured by NanoDrop ND-1000. RNA integrity was assessed by standard denaturing agarose gel electrophoresis. The Mouse 4 × 44 K Gene Expression Array v2 (Agilent Technology) with about 39,000+ mouse genes and transcripts represented, all with public domain annotations, was employed for genomic profiling. Sample labeling and array hybridization were performed according to the Agilent One-Color Microarray-Based Gene Expression Analysis protocol (Agilent Technology). Agilent Feature Extraction software (version 11.0.1.1) was used to analyze the acquired array images. Quantile normalization and subsequent data processing were performed with using the GeneSpring GX v11.5.1 software (Agilent Technologies). Differentially expressed genes were identified through Fold Change filtering.

### Preparation of recombinant proteins

PGRN was purified from conditioned medium of HEK-EBNA cells, which were stably transfected with C-terminal His-tagged human PGRN. Fusion proteins were affinity-purified using the ProBond purification system (Cat.K850-01, Life Technologies, Carlsbad, CA.) as described previously^12^. Protein purity was determined with SDS-PAGE and activity was measured with a TNFα blocking assay^28^.

### Differentiation of bone marrow-derived macrophages (BMDMs)

Bone marrow cells were isolated from mice tibia and femur and cultured with using L929-cell conditioned medium as a source of granulocyte/macrophage colony stimulating factor for 5 days to differentiate to BMDM^29^,^30^. The BMDMs were subsequently treated with TNF-α or IFN-γ for further experiments.

### Real-time Quantitative PCR analysis

1 × 10^5^ BMDMs isolated from C57BL/6 mice were stimulated with TNF-α (20 ng/ml) or IFN-γ (2.5 ng/ml) in the presence or absence of PGRN for 24 h. Total RNA was extracted from BMDMs by Trizol, and cDNA were synthesized by using SuperScript^®^ Reverse Transcriptase (Invitrogen). SYBR^®^ Green PCR Master Mix (Applied Biosystems) was employed in real-time PCR and the reaction was performed with StepOnePlus™ Real-Time PCR Systems (Applied Biosystems). The mRNA levels of target genes were normalized with 18 s RNA. The following sequence-specific primers were used for the real-time qPCR: 5′-tgtggagttcgagga accct-3′ and 5′-tgccttggctggtgctg-3′ for mouse CXCL9, 5′-ggatggctgtcctagctctg-3′ and 5′-tgagctagggaggacaagga-3′ for mouse CXCL10. The presence of a single specific PCR product was verified by melting curve analysis, and the experiments were repeated three times.

### Chemokine ELISA

CXCL10 and CXCL9 release from BMDMs was measured using a mouse-specific CXCL10 and CXCL9 ELISA kit (R&D Systems). BMDMs were isolated and differentiated from tibia and femur of WT and PGRN KO mice. 1 × 10^5^ cells/well BMDMs were plated in twelve-well plates and stimulated with TNF-α (20 ng/ml) or IFN-γ (2.5 ng/ml) in the presence or absence of PGRN (2.5 μg/ml) for 72 h and cell culture supernatants were harvested for ELISA assay according to the manufacturer’s recommendations.

### OXA-induced contact dermatitis model

Oxazolone (OXA)-induced contact dermatitis was induced as described^31^. In brief WT and PGRN−/− mice (n = 6 per group) were sensitized by a single application of 50 μl of 1.5% oxazolone (Sigma, MO, USA) in ethanol on the dorsal skin on Day 1. Seven days later, the inner and outer surfaces of ear were challenged with 20 μl of 1% OXA in a mixture of acetone and olive oil (4:1) on every other day over 7 days following initial OXA challenge. The mice were sacrificed after two weeks and ears were dissected and fixed for histology studies.

### Immunohistochemistry

The mice ear specimens were fixed in 10% formalin, dehydrated,cleared with dimethylbenzene, and then embedded in olefin. At least 4 consecutive 6-μm sections were obtained from the sagittal plane, and stained using hematoxylin and eosin (HE) for routine morphologic analysis. The skin samples were then incubated with anti-CXCL9 antibody (1:100 dilution; Santa Cruz Biotechnology) at 4 ^°^C overnight, followed by incubation with a horseradish peroxidase-conjugated secondary antibody for 60 min at room temperature. The signal was detected using the Vector Elite ABC Kit (Vector Laboratories, Burlingame, CA).

Ear thickness was measured at five different positions on each ear sample, and the average thickness for each mouse was calculated using ImageJ software based on the scale bar at 10X objective lens field. The data is presented as average levels with 6 mice per group. For quantification of CXCL9 staining, five images were taken for every mouse. CXCL9 positive staining area was determined by circularly bordering cells at 40X objective lens field using ImageJ. The percentage of CXCL9 positive area was determined by dividing the CXCL9 positive area by the total area under a 40X objective field.

## Results

### Gene expression analysis of CD4+ T cells isolated from WT and PGRN−/− mice

**Figure 1:**
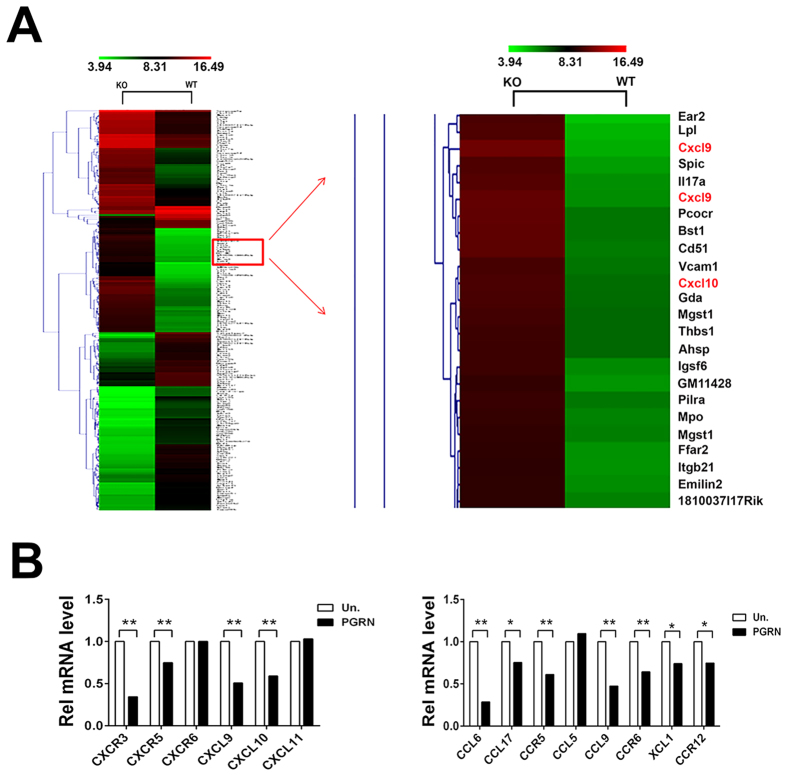
Genomic expression array in WT versus PGRN−/− cells. (**A**) RNA was isolated from CD4+ T cells from spleen of WT and PGRN−/− mice and gene expression analysis was performed. Differentially expressed genes were identified through Fold Change filtering. Hierarchical Clustering was performed to show the distinguishable gene expression profiling among samples (WT versus PGRN−/−). Each row corresponds to a gene and each column corresponds to an experimental sample. (**B**) PGRN inhibits the expression of several CC and CXC chemokines as identified in the microarray analysis. BMDM cells from WT mouse were treated with 200 ng/ml PGRN for 24 hrs, the mRNA level of chemokine were measured by quantitative PCR. This data represents results from three individual experiments. One-way ANOVA was used for statistical analysis (*p < 0.05, **p < 0.01).

Our previous studies have shown that PGRN has anti-inflammatory activities in RA and IBD^12^,^13^ and that recombinant PGRN induces T_reg_ population and IL-10 production by T cells^12^,^13^. Therefore, in order to investigate the molecular mechanisms underlying the anti-inflammatory activity of PGRN in immune response, we performed whole genome array with primary isolated CD4+ T cells from WT and PGRN−/− mice. Approximately 2600 genes were differentially regulated in PGRN−/− mice as compared to WT mice (Fig. 1A). Roughly 1200 of these differentially regulated genes were found to be upregulated in PGRN−/− mice. Interestingly, many chemokines and their receptors were significantly overexpressed in PGRN null mice, and among them, levels of CXCL10 and CXCL9 were most dramatically enhanced. In fact, genome analysis revealed a 64-fold increase in the expression of the CXCL9 gene in the PGRN−/− relative to WT mice ([KO] (raw) 3043, [WT] (raw) 47.8). CXCL10 was approximately 17 times more expressed as compared to WT mice levels ([KO] (raw) 24001, [WT] (raw) 1417.6). To validate the whole genome array data, we performed gain of function studies using recombinant PGRN; CXCL10 and CXCL9 were selected for further investigation due to their dramatic overexpression in PGRN−/− mice. We found that PGRN inhibited the expression of CC and CXC chemokines *in vitro* (Fig. 1B).

### Inhibition of CXCL9 and CXCL10 gene expression by recombinant PGRN

**Figure 2:**
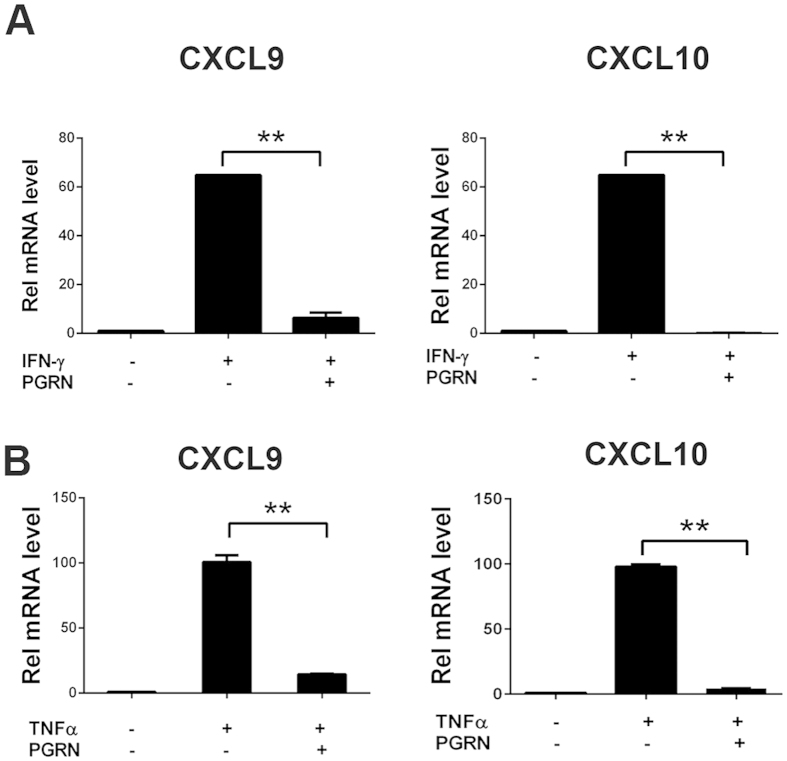
PGRN inhibits IFN-γ and TNFα activated gene expression of CXCL9 and CXCL10. BMDM cells from WT mouse were stimulated with 5 ng/ml of IFN-γ (**A**) or 20 ng/ml TNF-α (**B**) in presence of 200 ng/ml of PGRN, as indicated, for 24 hrs. Levels of CXCL9 and CXCL10 mRNA were measured by quantitative PCR. The data shown are representative of three independent experiments. One-way ANOVA was used for statistical analysis (**p < 0.01).

Previous studies have shown that CXCL10 and CXCL9 are mainly expressed by macrophages in RA synovium^27^. Therefore, we investigated the effect of PGRN on CXCL9 and CXCL10 in bone marrow derived macrophages (BMDMs). BMDMs from wild-type mice were stimulated with TNFα (20 ng/ml) or IFN-γ (2.5 ng/ml) in the presence or absence of PGRN (250 ng/ml) for 24 h. Total RNA was extracted and real-time quantitative PCR was performed. Untreated BMDMs produced small amounts of CXCL9 and CXCL10 mRNA, whereas stimulation with IFN-γ and TNFα led to a potent induction of CXCL9 and CXCL10 (60-fold increase by IFN- γ and 100-fold by TNFα). Addition of rPGRN protein almost completely blocked induction of CXCL9 and CXCL10 by IFN-γ and TNFα (Fig. 2A,B).

### Inhibition of CXCL9 and CXCL10 release by recombinant PGRN

**Figure 3:**
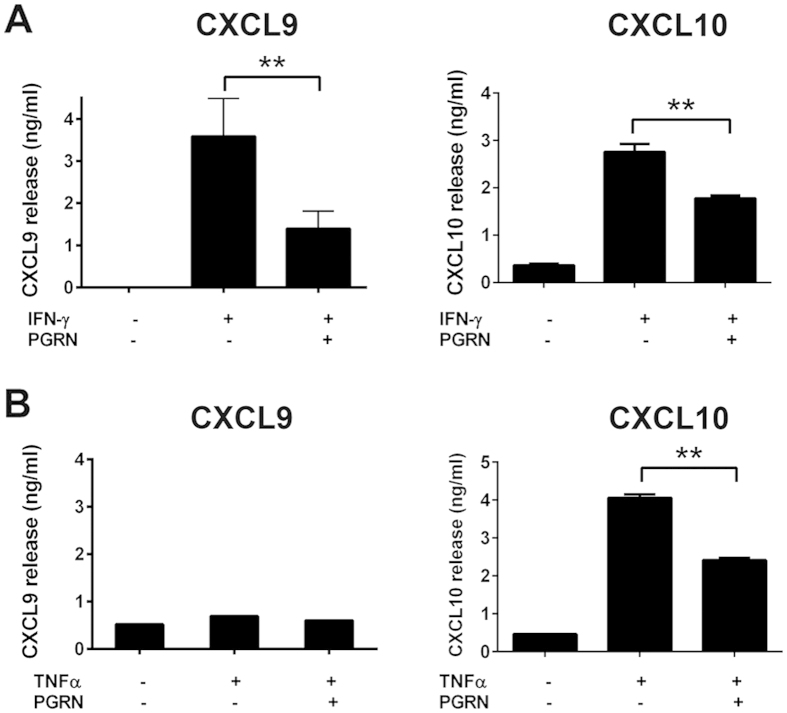
PGRN inhibits CXCL9 and CXCL10 release induced by IFN-γ or TNFα. BMDM cells were stimulated with 5 ng/ml IFN-γ (**A**) or 20 ng/ml TNF-α (**B**) in presence of 200 ng/ml of PGRN for 72 hrs. The cell culture mediums were collected to measure CXCL9 and CXCL10 release by ELISA kit. The data shown are representative of three independent experiments. One-way ANOVA was used for statistical analysis (**p < 0.01).

To further confirm results from our gene array and real-time PCR, we examined chemokine release induced by INF-γ and TNFα. BMDMs from WT mice were treated with either IFN-γ (20 ng/ml) or TNFα (100 ng/ml), with or without PGRN (200 ng/ml) for 72 hours, and release of CXCL9 and CXCL10 protein were assessed by ELISA. As shown in Fig. 3A IFN-γ dramatically induced both CXCL9 and CXCL10 release from BMDMs, and addition of rPGRN significantly inhibited release of both chemokines, which is consistent with real-time PCR results (Fig. 2A). In the TNFα treated group, release of CXCL10, but not CXCL9, was induced (Fig. 3B). Though the underlying mechanism remains unknown, our findings are consistent with a previous report on interactive stimulation of these inflammatory chemokines by IFN-γ and TNFα^32^.

### CXCL9 is highly upregulated in skin of OXA-induced contact dermatitis model

**Figure 4:**
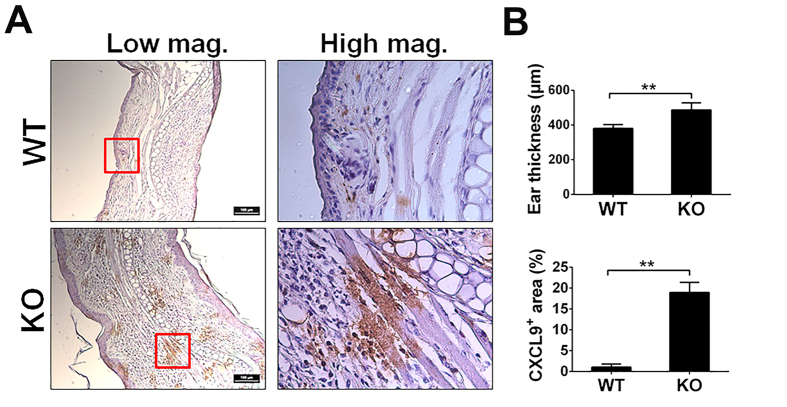
The expression of CXCL9 in ear of OXA-induced contact dermatitis. Contact dermatitis was induced on the ear of WT and PGRN KO mice by OXA (n = 6 group). After two weeks, mice were sacrificed and ear tissues were fixed for histology analysis. Ear thickness and CXCL9 expression were examined. (**A**) Immunohistochemistry staining of CXCL9 in ear. Tissue from WT and PGRN KO mice were stained with CXCL9 antibody and expression of CXCL9 is shown in brown. (**B**) Quantification of ear thickness (upper panel) and CXCL9 (lower panel) expression. Ear thickness is measured at five different positions on each ear sample and the average ear thickness for each mouse was calculated by Image J based on the scale bar. The data is presented as averaged thickness leveled across 6 mice per group. For of CXCL9 staining quantification, five images were taken for each sample under 40X objective lens field. And the positive staining were circled and calculated by Image J. The data is represented as ratio of CXCL9 positive area to total area of 40X objective lens field. One-way ANOVA was used for statistical analysis (**p < 0.01).

PGRN is reported to be elevated in skin inflammatory conditions and contributive to skin repair^33^,^34^. Upregulation of CXCL9 is also associated with inflammatory diseases. In fact, serum levels of CXCL9 were significantly higher in dermatitis patients than those found in normal controls^35^. Therefore, we investigated the expression pattern of CXCL9 in the skin of mice under inflammatory conditions using a previously established oxazolone (OXA)-induced contact dermatitis model^31^. The OXA-induced dermatitis model was established in WT and PGRN−/− mice, and the ear samples were assessed via histology. Ear tissue was found to be significantly thicker in the PGRN KO group compared with WT group (487 ± 40.5 μm in PGRN KO vs 380 ± 22.7 μm in WT) (Fig. 4), indicating more severe inflammation in PGRN null mouse. Expression of CXCL9 was also much higher in PGRN−/− mice and quantification analysis revealed that only 1% area produced CXCL9^+^ staining in WT mice while 18% area exhibited CXCL9^+^ staining in PGRN KO mice (Fig. 4). This result corresponds with *in vitro* data and supports our hypothesis that PGRN might regulate CXCL9 expression *in vivo* and that PGRN deficiency can lead to overexpression of CXCL9 and CXCL10, which in turn, promotes inflammatory conditions.

### PGRN inhibits CXCL10 and CXCL9 expression in TNFR1 dependent manner

**Figure 5:**
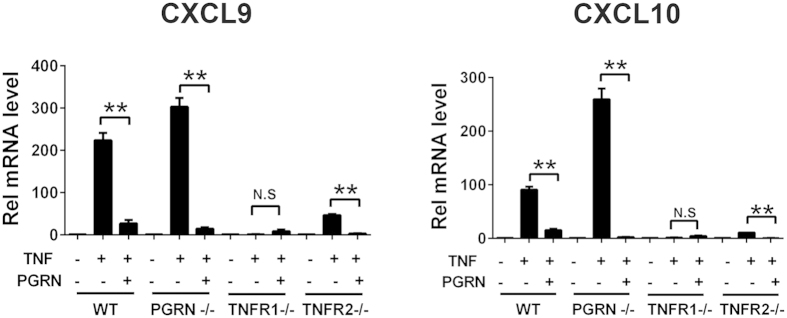
PGRN inhibits TNFα induced expression of CXCL9 and CXCL10 in a TNFR1 dependent manner. BMDM cells isolated from WT, PGRN−/−, TNFR1−/− and TNFR2−/− were stimulated with 20 ng/ml TNF-α in presence of 200 ng/ml of PGRN for 24 hrs. Expression of CXCL9 and CXCL10 were measured by quantitative PCR. Data gathered from experiments conducted in triplicate and one-way ANOVA was used for statistical analysis (**p < 0.01).

We have shown before that PGRN binds TNFR1 and TNFR2^12^,^28^. TNFR1 induces pro-inflammatory response upon binding with TNFα^36^ whereas TNFR2 is important for PGRN-mediated protective roles^37^,^38^,^39^. Therefore, to assess the importance of TNFR1 and TNFR2 for the anti-inflammatory effect of PGRN, we performed CXCL9 and CXCL10 gene expression assay in BMDMs from WT, PGRN−/−, TNFR1−/− and TNFR2−/− mice. BMDM cells were treated with TNFα, with or without PGRN, for 24 hours. In these cells, the expression of CXCL9 and CXCL10 were significantly induced after TNFα treatment and rPGRN protein almost completely blocked this effect in WT and PGRN KO mice. Interestingly, in TNFR1−/− BMDMs, TNF α failed to induce CXCL9 and CXCL10 expression and addition of rPGRN had no effect on expression of either chemokine, suggesting that TNF-induced CXCL9 and CXCL10 expression occurs mainly through TNFR1, and PGRN inhibition of CXCL9 and CXCL10 is likely TNFR1-dependent (Fig. 5A,B). In TNFR2−/− BMDMs, TNFα treatment still upregulated CXCL9 and CXCL10 expression, but to a much lower extent as compared with WT BMDMs. Recombinant PGRN protein consistently inhibited TNF-mediated induction of both chemokines, indicating PGRN’s inhibitory effect on chemokine expression doesn’t depend on TNFR2, but that TNFR2 is important to enhancement of TNFα mediated signaling, as loss of TNFR2 dramatically reduced TNF-α stimulation of CXCL9 and CXCL10 (Fig. 5A,B).

## Discussion

Chemokines and chemokine receptors form a complex network of molecules that play a vital role in leukocyte migration and activation. Deregulation of the chemokine network can cause abnormal accumulation of leukocytes at the inflammation site, which results in acute and chronic autoimmune inflammatory disorders such as RA, multiple sclerosis and IBD. Recently, chemokines have become promising therapeutic targets for effective treatment of inflammatory and autoimmune diseases^21^. Among the large chemokine and chemokine receptor families, CXCR3 and CXCR3-binding chemokines CXCL10 and CXCL9 and ITAC/CXCL11 are key players in the maintenance and amplification of the autoimmune disease. These proteins are not detectable under normal physiological conditions but are strongly induced in the pro-inflammatory milieu under pathologic conditions^40^,^41^,^42^. A recent study conducted human phase II clinical trials using anti-CXCL10 monoclonal antibody (MDX-1100) in RA patients and found that blocking CXCL10 significantly increased response rate at week 12 as compared to the placebo group^43^. Therefore, inhibition of chemokines such as CXCL10 could prove to be a great therapeutic strategy against RA. Our whole genome array showed significant upregulation of several chemokines including CXCL9 and CXCL10 in PGRN−/− mice compared to WT mice (Fig. 1A). We confirmed these observations using recombinant PGRN in *in vitro* gain of function studies and found that PGRN significantly inhibited CXCL9 and CXCL10 expression and release induced by IFN-γ and TNFα. Importantly, PGRN directly binds to TNFR1 and TNFR2 in many cell types^12^,^15^,^28^, here, we also found that TNF-induced CXCL9 and CXCL10 depends on TNFR1, and PGRN’s inhibitory effect upon CXCL10 and CXCL10 is mainly mediated through TNFR1 (Fig. 5). Intriguingly, PGRN also effectively inhibits IFN-γ-induced CXCL9 and CXCL10 expression. The underlying mechanism for this inhibition is unclear and needs further investigation, however, it is speculated that PGRN binds to TNFR, blocking TNF-**α** binding to the receptor, while simultaneously triggering a downstream signaling pathway, which may have crosstalk with the IFN-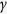-mediated pathway, leading to the inhibition of IFN-γ-induced CXCL9 and CXCL10 indirectly.

In addition, CXCL9, CXCL10 and CXCL11 bind to same membrane receptor, CXCR3, which was also upregulated in PGRN−/− mice according to our gene array data, and CXCR3 expression was inhibited by PGRN *in vitro* (Fig. 1B). Interestingly, CXCL11 was not identified in our whole genome array. Furthermore, PGRN did not inhibit CXCL11 in our *in vitro* gain of function studies. These three chemokines are unique in that each is induced by IFN-γ in a wide variety of cell types and each activates the same the chemokine receptor. However, it is now becoming clear that these chemokines exhibit unique expression patterns and may have nonredundant functions *in vivo*^21^. CXCL11 binds to CXCR3 with a higher affinity than CXCL9 and CXCL10^44^,^45^, but PGRN only inhibits CXCL9 and CXCL10, indicating PGRN inhibition effect on these chemokines is selective, and may be involved in fine-tuning the degree of chemokine function.

TNFα/TNFR interaction leads to pro-inflammatory signaling cascade and is a central mediator in several inflammatory diseases^9^,^14^,^31^,^46^. We report here that PGRN inhibited TNF-α induced expression and release of CXCL9 and CXCL10. These results reveal the downstream effect of PGRN on chemokines in addition to the previously shown, upstream antagonistic effect on TNFα. We have previously reported that PGRN−/− mice were susceptible to CIA and chemical-induced colitis and administration of recombinant PGRN reversed these inflammatory conditions^12^,^13^. We believe that PGRN tightly regulates CXCL9 and CXCL10 activity and loss of this regulation in PGRN−/− mice renders them more susceptible to inflammatory diseases such as RA and IBD.

The anti-inflammatory action of PGRN is exerted by its direct interaction with TNF receptors and by antagonizing TNF activity^12^,^14^,^31^,^47^,^48^. However, it is not clear which TNF receptor plays a major role in PGRN-mediated inhibition of chemokines. TNFR1 and TNFR2 have been shown to have shared and diverse functions which can be attributed to their location and activation by identical and unrelated molecules. TNFR1 is present in almost all cells, and TNFR1 cross-linking by TNFα leads to a pro-inflammatory response. TNFR2 is mainly found in T_reg_ cells and appears to be important for repair and homeostasis^49^. Therefore, we investigated the contributions of TNFR1 and TNFR2 signaling in chemokine inhibition by PGRN and found that this effect is mainly TNFR1 dependent. It has been previously reported that PGRN directly stimulates the numbers and function of T_reg_ under inflammatory conditions^12^,^16^. Therefore, it is possible that PGRN exerts its anti-inflammatory effect through TNFR1 and promotes T_reg_ activity through TNFR2. Therapeutic strategies such targeting PGRN, which has the potential to both inhibit inflammation and stimulate T_reg_ activity, might present a novel therapeutic concept to treat diverse inflammatory and autoimmune diseases.

In conclusion, this study demonstrates the critical role of PGRN in inhibition of chemokine expression and release. We also show that this effect is mainly dependent on the TNFR1. These results provide evidence of a new molecular mechanism of PGRN’s anti-inflammatory action. Importantly, these findings offer a new line of basic research upon which novel therapeutic strategies that exploit PGRN’s anti-inflammatory role in immune response for treatment of RA, IBD, and other auto-immune inflammatory diseases may be developed.
